# 954 Hydrofluoric Acid Fumes Associated with Electric Vehicle Lithium Ion Battery Fires

**DOI:** 10.1093/jbcr/iraf019.485

**Published:** 2025-04-01

**Authors:** Cherry Song, Michael Marano, Robin Lee, Christina Lee, Mukosolu Ndubisi, Mostafa Elbahrawy, Ayodeji Folarin

**Affiliations:** The Burn Center at Cooperman Barnabas; Cooperman Barnabas Medical Center; Cooperman Barnabas Medical Center; Cooperman Barnabas Medical Center; Rutgers New Jersey Medical School; Community Medical Center; Rutgers New Jersey Medical School

## Abstract

**Introduction:**

Lithium-ion batteries (LIB) are a common technology used in portable electronics, electric vehicles, and energy storage solutions. Electric cars and e-bikes have increased in popularity and demand, with a projection to grow in the future. One of the most recognized safety issues for LIB is thermal runaway. This process involves a rapid self-heating mechanism driven by internal exothermic reactions, leading to cell destruction and the release of toxic flammable gases, which poses a high risk of fire or explosion. The process may be initiated by disruption of battery integrity, internal failure, or mistreatment. The effects include release of gaseous hydrogen fluoride (HF) amongst other toxic chemicals. There are reports of hydrofluoric acid inhalation injury cases, however none have been linked to LIB fires. There should be a heightened awareness that electric car and e-bike fires emit these toxic fumes, and pose danger to patients who are trapped in an enclosed electric car space or an enclosed room where e-bikes are stored and charged.

**Methods:**

Literature search via Ovid, PubMed, EBSCOhost, and Google Scholar was performed. Keywords included lithium ion battery, lithium-ion battery fire, hydrofluoric acid burn, and HF inhalation injury. Search was filtered for the last 5 years and limited to publications in English.

**Results:**

There were no articles directly linking HF inhalation injury to LIB fire, although it is known that LIB produces HF fumes. There were a few reported cases of HF inhalational injury in factory workers at various metal factories. The fire departments have published educational materials for firefighters’ safety in situations where HF fumes are suspected. The CDC has made recommendations for the treatment of HF inhalation with nebulized calcium gluconate, however the success of this method has not been demonstrated.

**Conclusions:**

There is a need to raise awareness of possible HF inhalation injury in the setting of electric vehicle or e-bike fires in enclosed spaces. More studies should be performed regarding the etiology, diagnosis, and treatment of HF inhalation injury.

**Applicability of Research to Practice:**

In the setting of increasing electric car and bike use, more studies will need to be performed regarding the etiology, diagnosis, and treatment of HF inhalation injury.

**Funding for the Study:**

N/A

